# Development of an Acellular Tumor Extracellular Matrix as a Three-Dimensional Scaffold for Tumor Engineering

**DOI:** 10.1371/journal.pone.0103672

**Published:** 2014-07-29

**Authors:** Wei-Dong Lü, Lei Zhang, Chun-Lin Wu, Zhi-Gang Liu, Guang-Yan Lei, Jia Liu, Wei Gao, Ye-Rong Hu

**Affiliations:** 1 Department of Thoracic Surgery, Tumor Hospital of Shaanxi Province, Affiliated to the Medical College of Xi'an Jiaotong University, Xi'an, Shaanxi Province, PR China; 2 Department of Thoracic Surgery, Key Laboratory of Cancer Prevention and Therapy, Tianjin Lung Cancer Center, Tianjin Medical University Cancer Institute and Hospital, Tianjin, PR China; 3 Department of Thoracic and Cardiovascular Surgery, Second Xiangya Hospital of Central South University, Changsha, Hunan Province, PR China; University Hospital of Modena and Reggio Emilia, Italy

## Abstract

Tumor engineering is defined as the construction of three-dimensional (3D) tumors *in vitro* with tissue engineering approaches. The present 3D scaffolds for tumor engineering have several limitations in terms of structure and function. To get an ideal 3D scaffold for tumor culture, A549 human pulmonary adenocarcinoma cells were implanted into immunodeficient mice to establish xenotransplatation models. Tumors were retrieved at 30-day implantation and sliced into sheets. They were subsequently decellularized by four procedures. Two decellularization methods, Tris-Trypsin-Triton multi-step treatment and sodium dodecyl sulfate (SDS) treatment, achieved complete cellular removal and thus were chosen for evaluation of histological and biochemical properties. Native tumor tissues were used as controls. Human breast cancer MCF-7 cells were cultured onto the two 3D scaffolds for further cell growth and growth factor secretion investigations, with the two-dimensional (2D) culture and cells cultured onto the Matrigel scaffolds used as controls. Results showed that Tris-Trypsin-Triton multi-step treated tumor sheets had well-preserved extracellular matrix structures and components. Their porosity was increased but elastic modulus was decreased compared with the native tumor samples. They supported MCF-7 cell repopulation and proliferation, as well as expression of growth factors. When cultured within the Tris-Trypsin-Triton treated scaffold, A549 cells and human colorectal adenocarcinoma cells (SW-480) had similar behaviors to MCF-7 cells, but human esophageal squamous cell carcinoma cells (KYSE-510) had a relatively slow cell repopulation rate. This study provides evidence that Tris-Trypsin-Triton treated acellular tumor extracellular matrices are promising 3D scaffolds with ideal spatial arrangement, biomechanical properties and biocompatibility for improved modeling of 3D tumor microenvironments.

## Introduction

Pre-clinical research on cancer phenotype, aggressiveness and drug resistance is usually performed in two-dimensional (2D) *in vitro* cancer cell culture models or in xenograft animal models [1]. However, 2D cancer cell culture models do not provide a proper *in vivo* phenotype, due to the lack of a three-dimensional (3D) architecture for proper cell–cell and cell-matrix interactions. Tumor xenograft models can mimic tumor microenvironments in humans. Nevertheless, animals introduce many uncontrollable factors, including hemodynamics, host cells, endogenous growth factors and immune responses [2]. Moreover, monitoring of response to treatment for tumor xenograft models is expensive and difficult [3].

3D *in vitro* culture models are used in an effort to replicate the tumor specific cellular and matrix microenvironments and provide a significant alternative to both limitations of 2D monolayer culture and complex *in vivo* xenogeneic implantation approaches [1], [4]. 3D culture models help mimic cancer growth, progression and metastasis by providing a more controlled environment, in which growth factors, extracellular matrix (ECM) proteins and cell types are controlled [5]. Recently, some promising 3D tumor cell culture models have been developed and showed encouraging results [2], [6]–[9].

Tissue engineering approaches were employed to study tumor cells in 3D, which was defined as tumor engineering [10]. 3D scaffolds have been developed as platforms for study of *in vitro* cancer cell growth, proliferation, and migration. Scaffolds for 3D tumor cell cultures contain two main categories in the case of main components: synthetic and natural scaffolds. Synthetic polymers, including polyacrylamide (PA) [11], polystyrene (PS) [12], poly(ethylene glycol) (PEG) [13], poly(lactic acid) (PLA) [14] and poly(lactic-coglycolic acid) (PLGA) scaffolds [6], are specifically designed to replicate the *in vivo* tumor microenvironment. Synthetic scaffolds have the potential to resemble some characteristics of the natural microenvironment since they can be designed to function as needed. However, cell adhesion to synthetic scaffolds is poor due to the lack of *in vivo*-like structure and natural components.

Natural scaffolds include basement membrane proteins (Matrigel) [15], [16], collagen hydrogels [2], hyaluronic acid-based hydrogels [17], alginate [18] and chitosan-alginate composite scaffolds [19]. By culturing cancer cells with specific ECM constituents, it is possible to mimic some patterns of the *in vivo* cell-cell and cell-ECM interactions [20]. Nevertheless, if an improved *ex vivo* duplication of tumor performance is desired, it will be advantageous to more closely mimic the correct ECM composition, structure and biomechanical property of native tumors. These natural scaffolds can not fully reflect the native *in vivo* tumor microenvironment.

An acellular natural matrix may retain the biomechanical property and unique ECM composition and structure of native tissues, so as to serve as a platform for tissue engineering [21], [22]. Hence, we hypothesized that decellularized tumor tissues would serve as ideal scaffolds for tumor engineering. In this research, solid tumors derived from the A549 human pulmonary adenocarcinoma cell xenotransplatation model were employed and decellularized comparing the efficacy of four different protocols. Scaffolds with complete cellular removal were chosen for further evaluation of structure, biomechanical pattern and 3D cancer cell culture. The study aims were to construct tumor-derived 3D scaffolds with ideal spatial arrangement, biomechanical property and biocompatibility for *in vitro* tumor research in the future.

## Materials and Methods

### Ethics statement

The animal experimental procedures and protocols were approved by the Animal Care and Use Committee of Tumor Hospital of Shaanxi Province (Permit Number: SXCH2012014). Animal experiments and housing procedures were performed in strict accordance with the laboratory animal administration rules of the Ministry of Science and Technology of the People's Republic of China. All surgery was performed under 4% sodium pentobarbital (40 mg/kg) anesthesia, and all efforts were made to minimize suffering. Since tumor A549, MCF-7, SW-480 and KYSE-510 cell lines were not *de novo* cell lines, ethical committee approval was not required.

### Tumors

Human pulmonary adenocarcinoma A549 cells (purchased from the American Type Culture Collection, ATCC, Catalog #CCL-185) were cultured in Dulbecco's modified Eagle's medium (DMEM) nutrient mixture (Invitrogen Corp., Carlsbad, CA), containing 1% penicillin-streptomycin and 10% fetal bovine serum (FBS, Invitrogen Corp.) in a humidified 37°C, 5% CO_2_ incubator. The amplified cells were trypsinized and resuspended in culture medium, and subcutaneously injected into the left and right flanks (ca. 3×10^5^ cells for each side) of severe combined immunodeficiency (SCID) mice (6-week-old male mice, obtained from Beijing Experimental Animal Centre of the Chinese Academy of Sciences). The tumor volumes were measured every 10 days using a caliper and calculated according to Feldman et al. [23] with the following the formula: 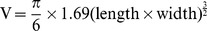. The measurements were ended on the 60th day of implantation (n = 6 for each time point). Gross and histological examinations were performed, and the 30-day implanted tumors were chosen for further treatment.

### Decellularization

Tumors retrieved at 30-day implantation were sliced into sheets (2 mm in thickness) with a pathological drawn knife (Kaixiu Trading Co., Ltd., Guangzhou, Guangdong, China) and sheared to disks (8–10 mm in diameter). Then the tumor sheets were decellularized with four procedures (group A–D) according to the protocols described by Yang et al. [24] and Deeken et al. [25], but with several modifications.

Group A (PAA group): Tumor sheets were decellularized with aqueous solution of 0.1% (v/v) peroxyacetic acid (PAA) and 4% (v/v) ethanol for 16 hours.

Group B (Trypsin-Triton group): Decellularization was carried out by 0.025% (wt/v) trypsin/0.02% (wt/v) EDTA (both Sigma Ltd., Poole, Dorset, UK) for 30 minutes, and followed by 0.5% (v/v) Triton X-100 (Amresco Inc., Solon, OH) for 48 hours.

Group C (Tris-Trypsin-Triton group): Tumor sheets were treated in hypotonic Tris buffer (10 mM Tris, 5 mM EDTA, pH 8.0) at 0°C overnight, and then incubated in hypertonic Tris buffer (50 mM Tris, 1M NaCl, 10 mM EDTA, pH 8.0) at 37°C for 24 hours. Finally, the treated tissues were subjected the decellularization procedure of group B.

Group D (SDS group): Tumor sheets were incubated in hypertonic Tris buffer containing 0.5% (wt/v) sodium dodecyl sulfate (SDS) at 37°C for 24 hours.

Samples for all groups were incubated in DNase I (20 U/mL)/RNase A (0.2 mg/mL) (both Invitrogen Corp.) containing 50 mmol/L MgCl_2_ for 24 hours. Except for the step of hypotonic Tris buffer treatment at group C, other steps were conducted under continuous shaking condition with 70 revolutions per minute (RPM) at 37°C. Aseptic phosphate-buffered saline (PBS) was employed after all treated procedures to remove the residual substances.

Gross examinations were performed after decellularization. One part of the treated samples and native controls were fixed in 2.5% glutaraldehyde for the scanning electron microscopy (SEM) examination (n = 3), or fixed in 10% phosphate-buffered formalin and subsequently embedded in paraffin for the light microscopic investigation (n = 3). Further parallel samples were used for porosity and biomechanical analysis. The other samples were lyophilized for 24 hours to dry weight and stored for further use.

### Cellular removal evaluation

For light microscopic examination, parallel 5 µm paraffin sections were routinely stained with hematoxylin–eosin (H&E) and Hoechst 33342. Immunohistochemical staining was performed using cytokeratin 7 (CK7) polyclonal antibody (1∶50 dilution, Sigma Ltd.) to identify tumor A549 cells and α-smooth muscle actin (α-SMA) monoclonal antibody (1∶200 dilution, clone 1A4, Sigma Ltd.) to detect mesenchymal cells. The avidin-biotinylated peroxidase complex method (Vectastain ABC kit, Vector Lab., Burlingame, CA) was used for the immunohistochemical staining. Slide-mounted tissue sections were washed with PBS, treated with 3% H_2_O_2_ in methanol for 5 minutes, predigested with 0.4% pepsin for 30 minutes at 37°C, preincubated with goat nonimmune serum for 30 minutes at 37°C, and incubated with CK7 or α-SMA antibody overnight at 4°C. The sections were then incubated with biotinylated horse anti-mouse/rabbit IgG (Vector Lab.) for 60 minutes at 37°C. After washing, the sections were treated with avidin-biotinylated horseradish peroxidase complex for 30 minutes at 37°C, and developed with 3-amino-9-ethylcarbazole (AEC, Vector Lab.). Sections were counterstained with hematoxylin for 5 minutes, and aqueously mounted in VectaMount AQ mounting medium (Vector Lab.). For the negative control, samples were incubated with goat nonimmune serum without the primary antibody.

For DNA content assay of lyophilized samples, DNA was extracted with the UltraClean tissue and cell DNA isolation kit (Mo Bio Laboratories, Inc., Carlsbad, CA), and DNA content of samples (n = 10) was quantified using the Quant-iT PicoGreen dsDNA Assay kit (Invitrogen Corp.). Briefly, samples were digested with DNA isolation solutions as described by the manufacturer's manual. Proteinase K digestion (final concentration of 0.5 mg/mL) was used in the procedure and tubes containing the tissue samples were vortexed for 10 minutes and incubated at 60°C for 30 minutes. The protein was precipitated and discarded. The remaining DNA was diluted with 1 mL TE buffer (10 mM Tris-HCl, 1 mM EDTA, pH 7.5), and then mixed with 1 mL of the aqueous working solution of Quant-iT PicoGreen reagent for 5 minutes in 96-well microplates, protected from light. Test solution was measured with a Spectrafluor microplate reader (Tecan Ltd., Sunrise, Austria) at an excitation wavelength of 480 nm and emission wavelength of 520 nm. The amounts of DNA were calculated against a λ DNA standard curve prepared at 10, 25, 50, 100, 250, and 500 ng/mL and expressed as ng/mg dry tissue weight.

### ECM component evaluation

Since samples in group C and D showed complete cellular removal, they were chosen for further ECM component testing, with the native samples used as controls. Ultrastructures were investigated with SEM examination (n = 3) as we previously described [26]. In brief, samples were fixed in 2.5% glutaraldehyde for 2 hours, postfixed in 1% OsO4 for 1 hour, briefly rinsed in distilled water, and dehydrated with graded ethanol. The specimens were then critical-point dried, sputter-coated with gold and examined with a Nova NanoSEM 230 microscope (FEI Co., Hillsboro, OR). Collagen fibrils were stained by Masson's trichrome technique and glycosaminoglycans (GAGs) were stained by Scott's alcian blue method.

Collagen and GAG contents (n = 10) were assayed as we previously reported [27]. Briefly, samples were hydrolyzed in 6 N HCl at 110°C for 15 hours. The produced hydroxyproline was oxidized by chloramine-T (1.4% w/v in acetate-citrate buffer, pH 6.0) and incubated in Ehrlich's reagent at 60°C for 20 minutes. The color density was quantified at 550 nm using a Beckman DU-600 spectrophotometer (Beckman Coulter Inc., Brea, CA). The hydroxyproline content of each sample was calculated using a standard curve from a graded concentration of L-4-hydroxyproline. Hydroxyproline content was converted to collagen content by assuming a factor of 12.5%. GAGs were extracted with papain type III solution (25 mg/mL, Sigma Ltd.) containing 0.1 M NaH_2_PO_4_, 5 mM EDTA, and 5 mM cysteine HCl at 60°C for 24 hours. Sulfated GAG content was determined spectrophotometrically at 590 nm wavelength after reaction with dimethylmethylene blue by using bovine chondroitin sulfate (Sigma Ltd.) as a standard. Collagen and GAG contents were expressed as µg/mg dry tissue weight.

### Scaffold porosity

The porosities of native and decellularized tumor sheets for group C and D (n = 6) were studied using the solvent displacement method reported by Zhang and Ma [28]. Since tumor sheets were not large enough, five pieces of them in the same group were employed as one sample. In brief, a dry sample (five pieces of tumor sheets) was immersed into a graduated cylinder filled with a known volume (V1) of absolute ethanol, and then the cylinder was placed in vacuum for 30 minutes to enable complete penetration of ethanol into the pores of the scaffold. The total volume of sample-included ethanol was recorded as V2. The sample was removed from the cylinder and the volume of residual ethanol was recorded as V3. The porosity (%) was calculated as follows:
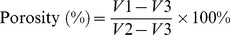
Where (V1–V3) represents volume of ethanol retained in the sample and (V2–V3) represents total volume of the sample.

### Tissue biomechanical property

The uniaxial tensile tests of native and decellularized tumor sheets for group C and D (n = 10) were measured with a Lloyd LRX material testing machine (Lloyd Instruments Ltd., Fareham, Hampshire, UK) as we previously reported [27]. Briefly, the specimens (5 mm in width and 8 mm in length) for wet tests were prepared by soaking them in PBS at room temperature for 8 hours to reach the fully swollen state, and then stress-loaded with a rate of 0.05 mm/s at 23°C and humidity of 50% until rupture. Sandpapers were attached to the grips of tensile test to prevent slipping. The elastic modulus (E) of samples was obtained from the linear region of the stress-strain curve for each specimen between 5–10% as follows:
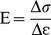
Where Δσ is the subtraction of stress values and Δε is the subtraction of strain displacement values for the linear region.

### MCF-7 cell culture

Human breast cancer MCF-7 cell line was purchased from ATCC. Growth factor reduced Matrigel matrix was purchased from BD Biosciences Company (San Jose, CA). For Matrigel samples, 120 µL fully supplemented DMEM nutrient media were mixed with 30 µL Matrigel matrix at 37°C for 60 minutes to form a gel for further use. The lyophilized samples in group C and D were sterilized by γ-ray irradiation (25 KGy). Before use, samples were sheared to disks (5 mm in diameter) and rehydrated in culture media for 16 hours.

MCF-7 cells were cultured onto the acellular scaffolds in group C and D for 1, 4, 7, 10 and 13 days, with the 2D culture and cells cultured onto the Matrigel scaffold used as controls (n = 12 for each time point). For the 2D culture group, cells were seeded in 24-well plates at 5×10^4^ cells in 1 mL fully supplemented DMEM nutrient media per well. For the Matrigel control and acellular scaffolds in group C and D, 5×10^4^ cells in 50 µL culture media were seeded onto each scaffolds and incubated at 37°C for 60 minutes, and then 1 mL fully supplemented DMEM nutrient media, containing 1% penicillin-streptomycin and 10% FBS, was added to each well for further culture. Cell culture medium was carefully changed every other day.

### Cell viability assay

The viability of MCF-7 cells cultured on 2D, the Matrigel control and the acellular scaffolds in group C and D were quantitatively determined by the 3-(4,5-dimethylthiazol-2-yl)-2,5-diphenyltetrazolium bromide (MTT) assay (Vybrant MTT Cell Proliferation Assay Kit, Invitrogen Corp.). Five of twelve samples (n = 5) for each time point were chosen for MTT assay. Briefly, 20 µL of MTT stock solution (5 mg/mL) was added to each well and incubated at 37°C for 4 hours, and then the medium was aspirated and 150 µL of dimethyl sulfoxide (DMSO) was added to each well. After gently shaking for 10 minutes, the MTT dye solution was transferred into 96-well assay plates and optimal density (OD) value for absorbance was measured at a wavelength of 570 nm in a microplate spectrophotometer (Bio-Tek Instruments Inc., Winooski, VT).

### Growth factor secretion with ELISA assays

Growth factor (interleukin 8 (IL-8), basic fibroblast growth factor (bFGF), and vascular endothelial growth factor (VEGF)) secretion was determined by ELISA kits (R&D Systems Inc., Minneapolis, MN) following the manufacturer's protocol. At 10-day culture, cell culture media were removed and cells for 2D and 3D culture were incubated in low serum media containing 1% FBS for 24 hours. Seven of twelve samples (n = 7) were chosen for the growth factor secretion evaluation. Matrigel and acellular scaffolds were discharged from the media for further histological evaluation, and the residual media and media for 2D culture were used to detect growth factor secretion.

### Histological evaluation of cell culture

Light microscopy investigation followed conventional procedures. To visualize live imaging of cells on culture, 2D cultured cells or cell seeded scaffolds (n = 3) were incubated in PBS containing 5 mM carboxyfluorescein diacetate succinimidyl ester (CFDA-SE) and 0.5 mg/ml propidium iodide (PI) at 37°C for 10 minutes, rinsed twice with PBS and finally observed with a fluorescent microscopy (E600, Nikon Corp., Tokyo, Japan). The cell-seeded acellular scaffolds in group C and D (n = 4) were fixed in 10% phosphate-buffered formalin and subsequently embedded in paraffin. H&E staining of cross-sections was used to evaluate the repopulation of tumor cells in the scaffolds.

### Other cell lines cultured within the Tris-Trypsin-Triton treated scaffold

Human colorectal adenocarcinoma cell line SW-480 and human esophageal squamous cell carcinoma cell line KYSE-510 were purchased from ATCC. A549, SW-480 and KYSE-510 cells have been implanted within the Tris-Trypsin-Triton treated scaffold, with the 2D monolayer culture used as a control. The viability of cultured A549, SW-480 and KYSE-510 cells were quantitatively determined by the MTT assay at 1-, 4-, 7-, 10- and 13-day culture (n = 5 for each time point).

### Statistical analysis

Independent experiments were performed three times for each condition tested *in vitro*, unless otherwise stated. Data were expressed as mean value ± standard deviation (SD). Statistical analysis of differences between multiple groups was performed using the one-way analysis of variance (ANOVA) followed by Tukey's multiple comparison tests. Statistical analyses were performed with SPSS (version 18.0, SPSS Inc., Chicago, IL). A value of p<0.05 was considered statistically significant.

## Results

### Tumor growth patterns

**Figure 1:**
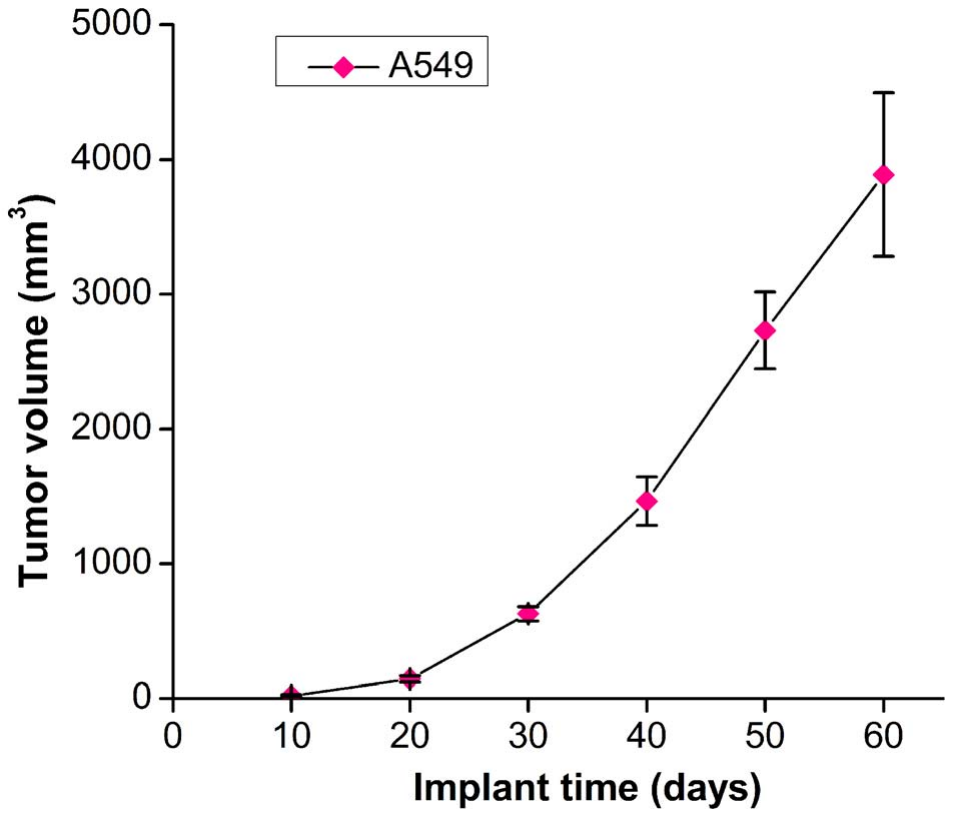
Growth curve of A549 cell derived tumors. Human pulmonary adenocarcinoma A549 cells were implanted in severe combined immunodeficiency (SCID) mice to form solid tumors. Note tumor volumes increased over time, with about 600 mm^3^ at 30-day implantation and about 4000 mm^3^ at 60-day implantation.

As shown in Figure 1, the tumor volumes increased with an extended implant time, with about 600 mm^3^ and 4000 mm^3^ at 30- and 60-day implantation respectively. Tumors were unilobate without necrosis at 30-day implantation, but tumors presented lobulation and necrosis after 30-day implantation, more notably at 60-day implantation. So tumors retrieved at 30-day implantation were chosen for further treatment.

### Cellular removal evaluation for native, PAA, Trypsin-Triton, Tris-Trypsin-Triton and SDS group

**Figure 2:**
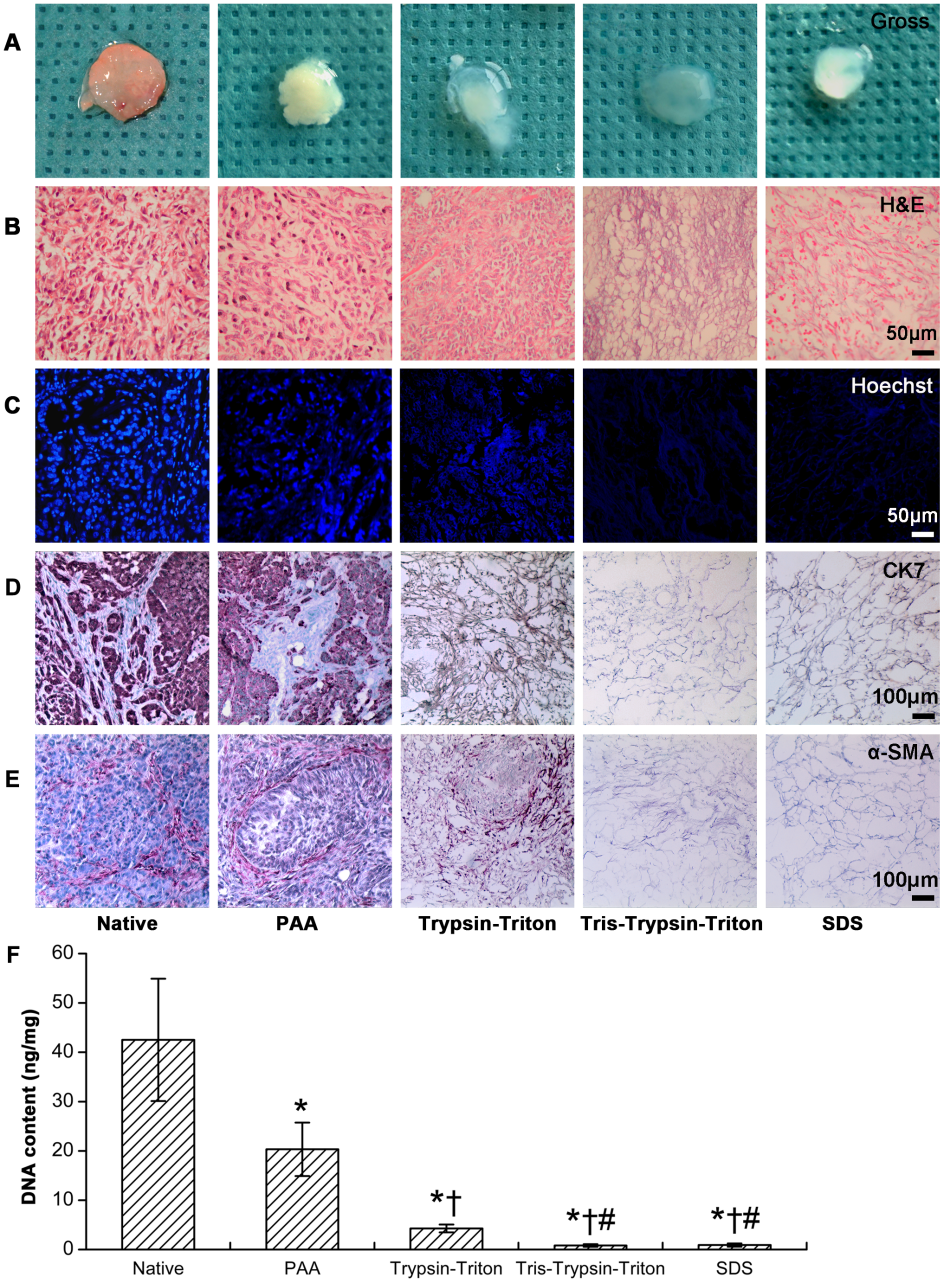
Cellular removal evaluation for PAA, Trypsin-Triton, Tris-Trypsin-Triton and SDS treated samples compared with the native samples. (A) Gross appearance showing solid and opaque for native and PAA treated tumor samples but translucent for Trypsin-Triton, Tris-Trypsin-Triton and SDS treated samples. (B) Hematoxylin–eosin (H&E) and (C) Hoechst staining showing complete cellular removal for Tris-Trypsin-Triton treated or SDS treated samples compared with samples of the other three groups. (D) Immunohistochemical staining for CK7 and (E) α-SMA showing complete removal of epithelial and mesenchymal cell components for Tris-Trypsin-Triton treated or SDS treated samples compared with samples of the other three groups. (F) DNA content analysis of native, PAA, Trypsin-Triton, Tris-Trypsin-Triton and SDS treated tumor samples. All presented results were mean ± SD (n = 10, *p<0.05 versus the native group; †p<0.05 versus the PAA group; #p<0.05 versus the Trypsin-Triton group).

Gross appearance in Figure 2A showed that native and PAA treated tumor sheets were solid and opaque, but Trypsin-Triton treated, Tris-Trypsin-Triton treated and SDS treated samples were translucent.

H&E staining in Figure 2B showed normal tumor structures and components for native tumor sheets, incomplete cellular removal for PAA treated or Trypsin-Triton treated samples, and complete cellular removal for Tris-Trypsin-Triton treated or SDS treated samples.

Hoechst staining in Figure 2C showed PAA treated and Trypsin-Triton treated samples had a decreased staining intensity of cell nuclei compared with the native tumors. Tris-Trypsin-Triton treated and SDS treated samples showed no cell nuclei staining.

Immunohistochemical staining in Figure 2D showed anti-CK7 antibody labelled tumor cells but not mesenchymal cells within native tumor sheets. Conversely, Figure 2E illustrates anti-α-SMA antibody mediated staining of mesenchymal cells but not tumor cells within native tumor sheets. PAA or Trypsin-Triton treated samples were stained positively for CK7 and α-SMA, but Tris-Trypsin-Triton treated or SDS treated samples were not stained by CK7 or α-SMA specific antibodies.

### DNA content assay for native and the four decellularized groups

DNA content assay shown in Figure 2F showed that the four decellularized groups had significant DNA decrease compared with the native group (all p<0.05). DNA content of Trypsin-Triton group was less than the PAA group (p<0.05). DNA contents of Tris-Trypsin-Triton group and SDS group were comparable (p>0.05), but less than the PAA group and the Trypsin-Triton group (all p<0.05). For their mean values compared with the native group, the residual DNA contents were 47.80% for PAA group, 10.04% for Trypsin-Triton group, 1.93% for Tris-Trypsin-Triton group and 2.14% for SDS group.

### ECM structures and components for native, Tris-Trypsin-Triton treated and SDS treated samples

**Figure 3:**
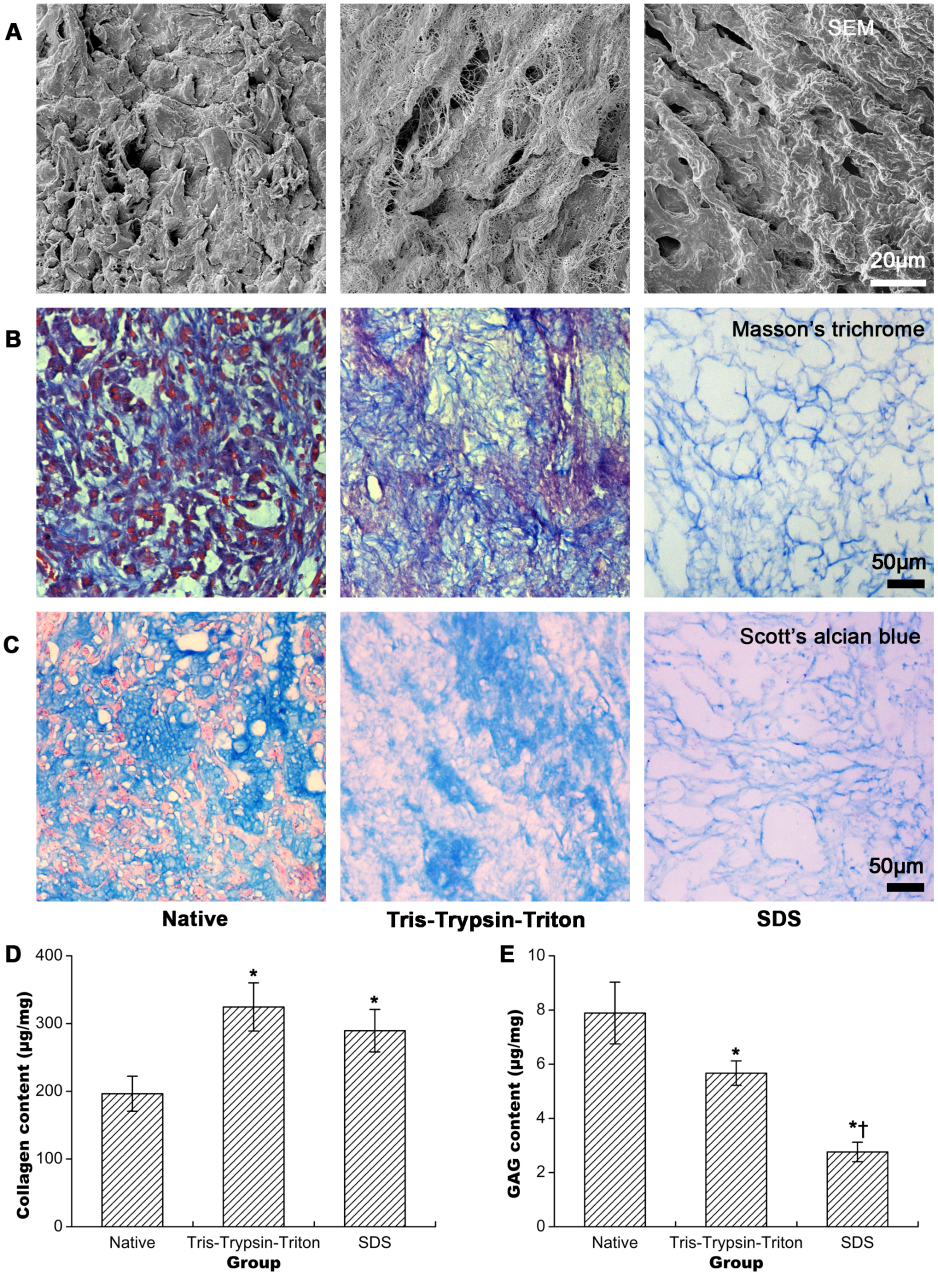
Extracellular matrix (ECM) structure and component investigation of native, Tris-Trypsin-Triton treated and SDS treated tumor sheets. (A) Scanning electron microscopy (SEM) examination for Tris-Trypsin-Triton treated and SDS treated samples showing lack of cellular components and having more open spaces than the native samples. (B) Collagen fibril stained by Masson's trichrome showing similar distribution of collagen fibrils for Tris-Trypsin-Triton treated samples but more sparse distribution of collagen fibrils for SDS treated samples compared with the native samples. (C) Glycosaminoglycan (GAG) staining with Scott's alcian blue presenting mostly retaining of GAGs for Tris-Trypsin-Triton treated sheets but less GAGs for SDS treated sheets compared with their native counterparts. (D) Collagen and (E) GAG content analysis of native, Tris-Trypsin-Triton treated and SDS treated tumor sheets. All presented results were mean ± SD (n = 10, *p<0.05 versus the native group; †p<0.05 versus the Tris-Trypsin-Triton group).

SEM examination in Figure 3A depicted compact cells on the surface of native tumor sheets. Tris-Trypsin-Triton and SDS treated samples lacked cellular components and had more open spaces than the native samples. Moreover, Tris-Trypsin-Triton treated samples displayed more micro-pores but less large porous structures than the SDS treated samples.

Masson's trichrome staining in Figure 3B showed compact collagen fibrils for native tumor samples. Tris-Trypsin-Triton treated samples had similar distribution of collagen fibrils compared to the native tumor samples, but SDS treated samples had more sparse distribution of collagen fibrils than the native and Tris-Trypsin-Triton treated samples.

Scott's alcian blue staining in Figure 3C presented plentiful distribution of GAGs for native tumor sheets. Tris-Trypsin-Triton treated samples retained most of GAGs, and SDS treated samples had less GAGs compared with their native counterparts.

Collagen and GAG content assays in Figure 3D and 3E showed collagen content increased but GAG content decreased for Tris-Trypsin-Triton treated or SDS treated samples compared with their native counterparts (all p<0.05). GAG content of SDS treated samples was less than that of Tris-Trypsin-Triton treated samples (p<0.05).

### Porosities for native and the two acellular tumor sheets

**Figure 4:**
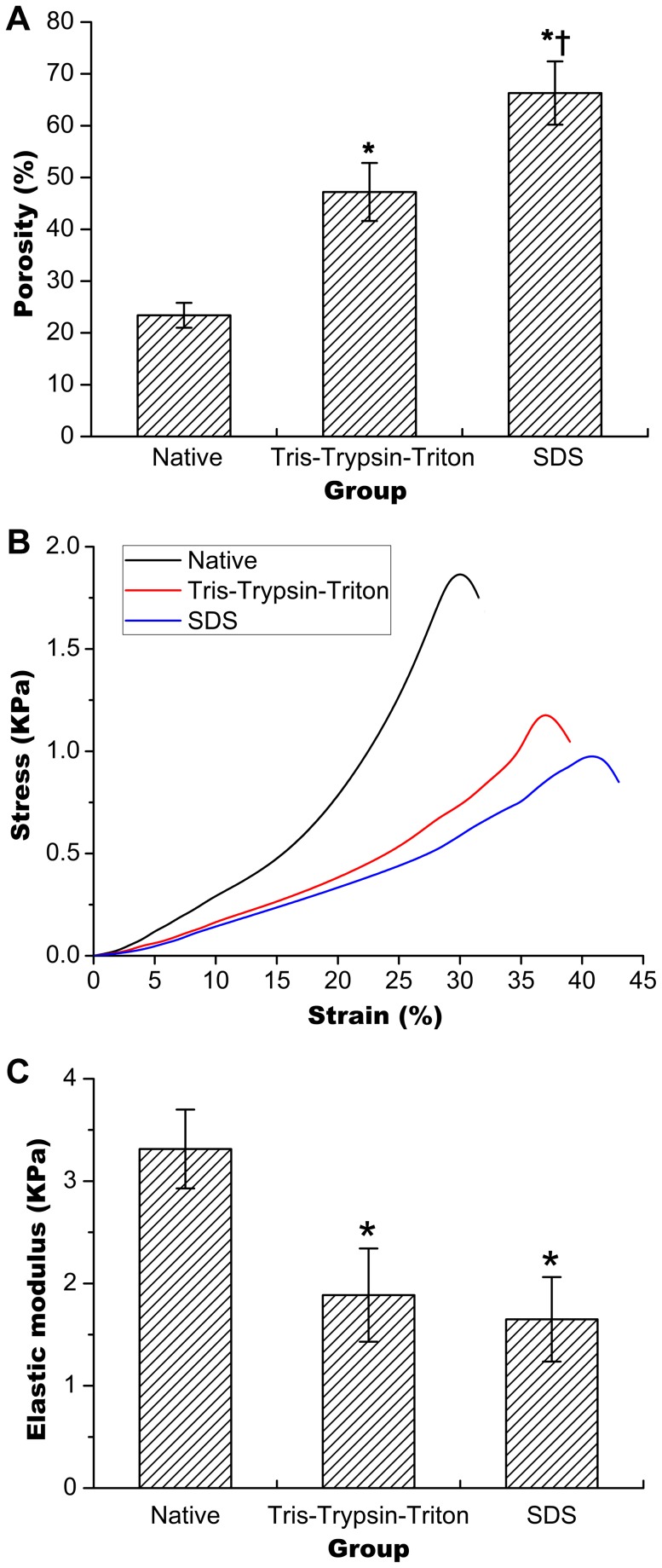
Porosities and biomechanical tests of native, Tris-Trypsin-Triton treated and SDS treated tumor sheets. (A) Increase of porosity for Tris-Trypsin-Triton treated or SDS treated samples compared with the native samples. (B) Separation of stress-strain curves for the three groups. (C) Less elastic moduli shown for Tris-Trypsin-Triton treated or SDS treated tumor sheets compared with the native samples. All presented results were mean ± SD (n = 6 for porosity test and n = 10 for biomechanical test, *p<0.05 versus the native group; †p<0.05 versus the Tris-Trypsin-Triton group).

Figure 4A shows porosity of Tris-Trypsin-Triton treated or SDS treated samples was greatly increased compared with their native counterparts (both p<0.05). Porosity of SDS treated samples was larger than that of Tris-Trypsin-Triton treated samples (p<0.05).

### Biomechanical property for native and the two acellular tumor sheets

Figure 4B shows separation of stress-strain curves for Tris-Trypsin-Triton treated and SDS treated tumor sheets compared with their native counterparts. Figure 4C presents that the elastic moduli of Tris-Trypsin-Triton treated or SDS treated tumor sheets were less than that of their native counterparts (both p<0.05). SDS treated tumor sheets had comparable elastic moduli compared with the Tris-Trypsin-Triton treated samples (p>0.05).

### Effects of 2D and 3D culture on MCF-7 cell viability and growth factor secretion

**Figure 5:**
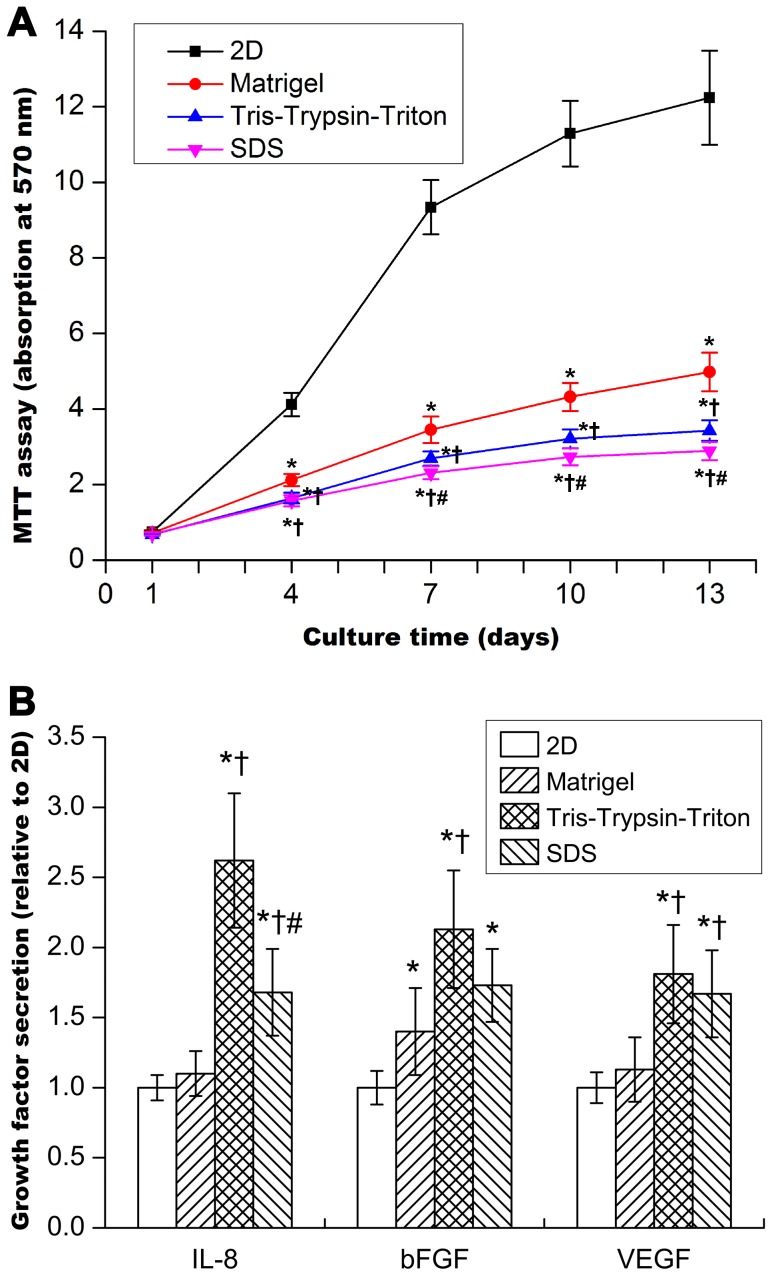
Cell viability and growth factor secretion assays for breast cancer MCF-7 cells. (A) Cell viability detected by MTT assay for MCF-7 cells cultured in 2D, Matrigel, Tris-Trypsin-Triton and SDS group over time. (B) Growth factor (IL-8, bFGF, and VEGF) secretion determined by ELISA assay for MCF-7 cells cultured in 2D, Matrigel, Tris-Trypsin-Triton and SDS group at 10-day culture. Graph represents mean ± SD of three independent experiments (n = 5 for MTT assay and n = 7 for growth factor secretion assay,*p<0.05 versus the 2D culture; †p<0.05 versus the Matrigel culture; #p<0.05 versus the Tris-Trypsin-Triton group).

Figure 5A presents similar cell viability for 2D, Matrigel, Tris-Trypsin-Triton and SDS group at 1-day culture (all p>0.05). Tris-Trypsin-Triton treated sheets and SDS treated sheets had similar cell viability at 4-day culture (p>0.05). At the time point of 7-, 10- and 13-day culture, 2D culture had the most cell viability, Matrigel group had the second most cell viability, and SDS group had the least cell viability. Cell viability gradually increased from 1-day to 10-day culture of the four groups. Cell viability at 13-day culture was similar to at 10-day culture of the four groups (all p>0.05).

Figure 5B shows growth factor (IL-8, bFGF, and VEGF) secretion assay for the 2D, Matrigel, Tris-Trypsin-Triton and SDS group at 10-day culture. With regard to cytokine secretion from cells seeded on decellularized matrices prepared by different methods, the preparation method was significantly influential. In particular, IL-8, bFGF and VEGF secretion was consistently greatest when cells were seeded on matrices prepared by Tris-Trypsin-Triton treatment; with levels of cytokine secretion approximately twice those observed in 2D monolayer or 3D matrigel control cultures.

### Histological performance for 2D and 3D culture

**Figure 6:**
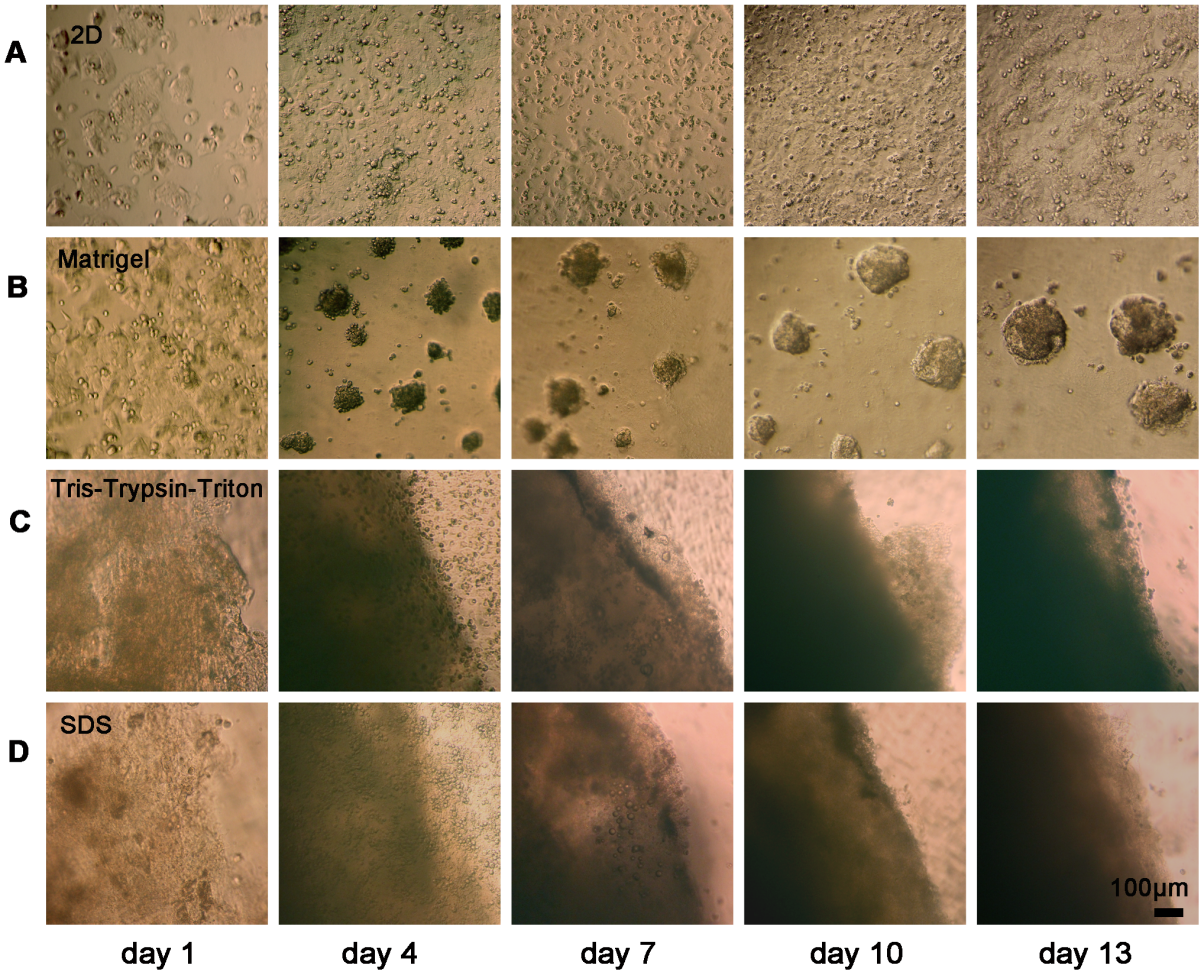
Representative light microscopy of MCF-7 cells cultured in 2D and different 3D scaffolds over time. (A) Representative images of MCF-7 cells grown in 2D culture, showing more and denser cells over time. (B) MCF-7 cells cultured in the Matrigel group, showing formation and enlargement of multicellular tumor spheroids (MCTS) over time and the growth pattern looked like the classic “growth on top” method. (C) MCF-7 cells cultured in the Tris-Trypsin-Triton group, showing gradual decrease of the light transmittance of scaffolds. (D) MCF-7 cells cultured in the SDS group, also showing gradual decrease of the light transmittance of scaffolds, but presenting more transmittance of light at each time point compared with the Tris-Trypsin-Triton treated scaffolds.

Figure 6 presents light microscopy of MCF-7 cells cultured in 2D, Matrigel, Tris-Trypsin-Triton treated and SDS treated scaffolds over time. For 2D culture (Figure 6A), cells were sparsely distributed at 1-day culture, and cells became more and denser at the later culture. Since it is transparent for Matrigel, small cell clusters can be observed at 1-day timeframe (Figure 6B). Multicellular tumor spheroids (MCTS) were formed at 4-day culture and enlarged continually over time. When cells cultured in Tris-Trypsin-Triton treated and SDS treated scaffolds (Figure 6C and 6D), translucent scaffolds were observed at 1-day culture. At 4-, 7-, 10- and 13-day culture, light transmittance of scaffolds gradually decreased and that of the Tris-Trypsin-Triton group is less than the SDS group at each time point.

**Figure 7:**
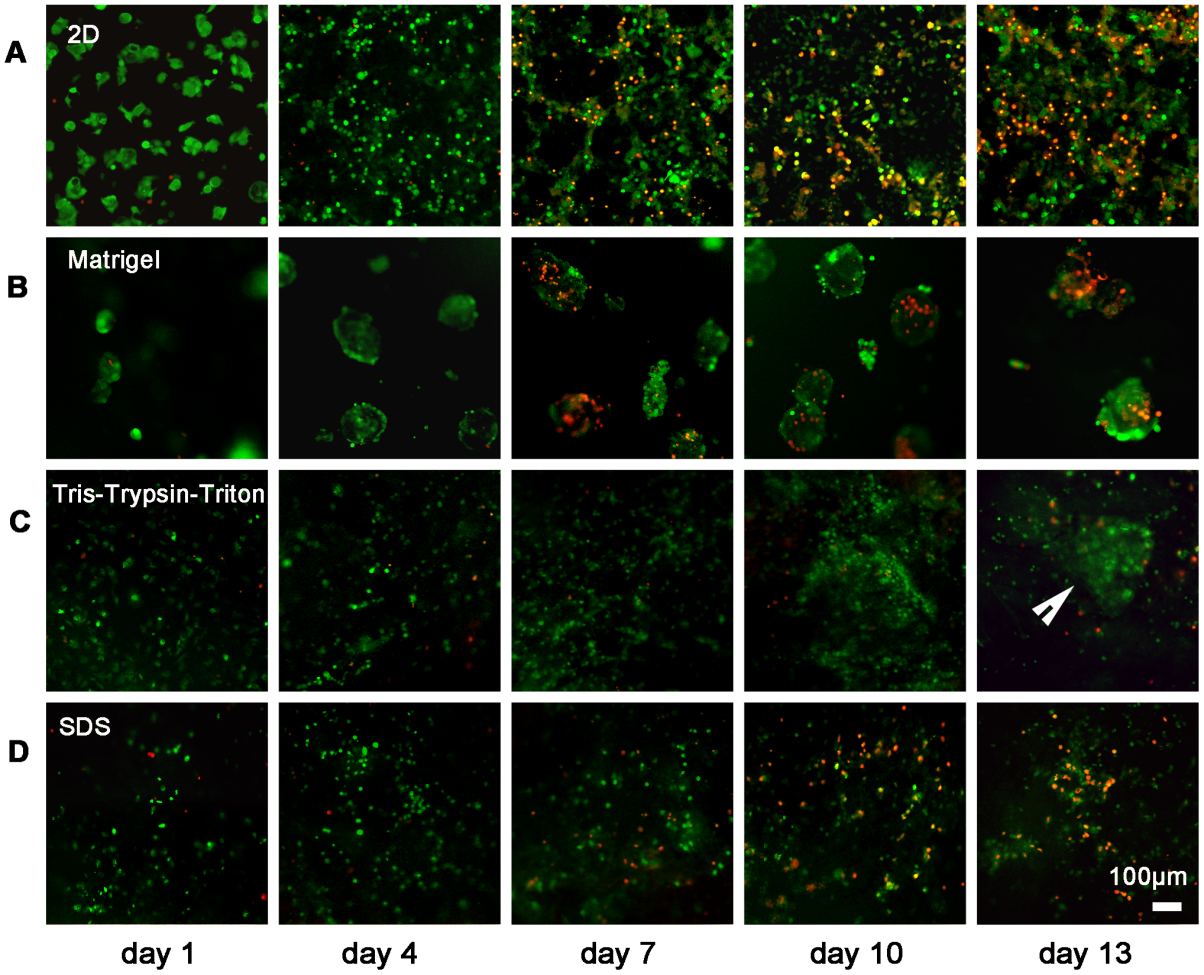
Representative fluorescence microscopic images of MCF-7 cells double-stained by CFDA-SE and PI for 2D and different 3D scaffolds over time. (A) Representative photomicrographs of MCF-7 cells grown in 2D culture, showing great increasing of dead cells from 4- to 13-day culture. Note CFDA-SE stained viable cells green and PI stained dead cells red. (B) MCF-7 cells cultured in the Matrigel group, showing distribution of dead cells in the core of MCTS. (C) MCF-7 cells cultured in the Tris-Trypsin-Triton group, showing higher proportion of live cells at 7-, 10- and 13-day culture and formation of cell clusters at 13-day culture (arrowhead). (D) MCF-7 cells cultured in the SDS group, showing more dead cells but less live cells compared with the Tris-Trypsin-Triton treated scaffolds at 7-, 10- and 13-day culture.

Figure 7 shows green-stained live cells which were visible with only a small number of red-stained dead cells for the four groups at 1-day timeframe. For 2D culture, live cells increased obviously from 1- to 4-day culture, and dead cells increased greatly from 4- to 13-day culture (Figure 7A). For cells cultured in Matrigel scaffolds, dead cells distributed in the core of formed MCTS (Figure 7B) at 7-, 10- and 13-day culture. For cells cultured in Tris-Trypsin-Triton treated and SDS treated scaffolds (Figure 7C and 7D), more live cells but less dead cells presented for Tris-Trypsin-Triton treated scaffolds compared with the SDS treated scaffolds at 7-, 10- and 13-day culture. Particularly, MCTS-like structure of cell clusters appeared when cells were cultured in the Tris-Trypsin-Triton treated scaffolds at 13-day culture (Figure 7C).

**Figure 8:**
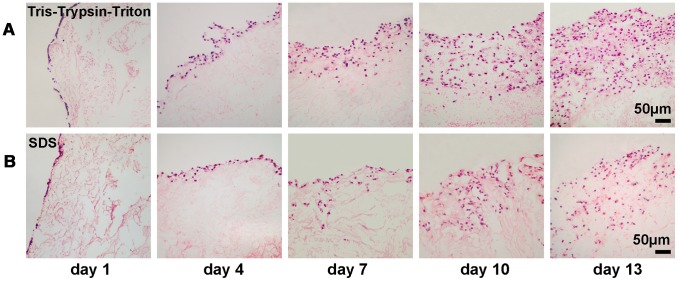
H&E-stained histological cross-sections of MCF-7 cells repopulated in the two acellular scaffolds over time. (A) MCF-7 cells cultured in the Tris-Trypsin-Triton group, showing increase of cell numbers and infiltration depths over time. (B) MCF-7 cells cultured in the SDS group, showing less cell numbers and infiltration depths compared with the Tris-Trypsin-Triton treated scaffolds at 7-, 10- and 13-day culture.

Histological cross-sections stained by H&E showed cell number and infiltration depth for Tris-Trypsin-Triton and SDS group increased over time (Figure 8). Tris-Trypsin-Triton treated sheets and SDS treated sheets had similar cell repopulation at 1- and 4-day culture. But at 7-, 10- and 13-day culture, Tris-Trypsin-Triton group had a greater number of cells and deeper infiltration distances than the SDS group.

### Repopulation performance of other cell lines within the Tris-Trypsin-Triton treated scaffold

A549, SW-480 and KYSE-510 cells did not all behave in the same way, with MCF-7-like behavior for A549 and SW-480 cells (Figure S1A and S1B) but slower repopulation by KYSE-510 cells consistent with their innately slower proliferation rate in monolayer culture (Figure S1C).

## Discussion

In this study, solid tumors derived from the A549 pulmonary adenocarcinoma cell xenotransplatation model were used as material sources for tumor engineering. To our knowledge, this is one of the first studies to use an acellular tumor extracellular matrix (ECM) as a scaffold for tumor engineering. Since tumors at 30-day implantation were large enough for further treatment and free of necrosis, it was chosen as the time point for tumor retrieval. We have also derived ECM from the KYSE-510 cell (human esophageal squamous cell carcinoma cell line) xenotransplatation model. The growth rate of tumors for KYSE-510 cells was much slower than A549 cells (Figure S2). Because of an impractical slow tumor growth rate, the KYSE-510 cell derived ECM was abandoned for further evaluation and application.

Four procedures were employed to decellularize the native tumor sheets to get cell-free tumor ECMs. The optimal procedure for decellularization should completely remove the cellular components while keep the ECM structure and component intact [21]. PAA was successfully used to decellularize porcine bladder and small intestine submucosa, owing to effective disruption of cell membranes and nucleic acids [29], [30]. Herein, cell nuclei were visible after decellularizing the tumor sheets with combination of PAA and DNase I/RNase A. Positive staining for both CK7 and α-SMA demonstrated incomplete removal of cellular components from tumor epithelial cells and mesenchymal cells. DNA content assay showed residual DNA content was 47.80% of the native tumor sheets, further implying incomplete cellular removal. As a result, PAA treatment was abandoned for decellularization of tumor sheets. Decellularization of bovine jugular vein conduits with combination of trypsin/EDTA, Triton X-100 and DNase I/RNase A led to complete cellular removal in our previous study [27], [31]. But in this study, light microscopic examination and DNA content assay confirmed incomplete decellularization of tumor sheets. When pretreated with hypotonic and hypertonic Tris buffer, and subsequently treated with trypsin/EDTA, Triton X-100 and DNase I/RNase A, tumor sheets had complete cellular removal. SDS effectively removed cellular components from heart valves, adipose tissues and urinary bladder [24], [32]–[34]. The present investigation demonstrated complete cellular removal for tumor sheets with combination of SDS and DNase I/RNase A. Therefore, subsequent studies focused on the Tris-Trypsin-Triton group and SDS group.

ECM components, structures, porosities and biomechanical patterns were investigated to determine whether these properties were altered due to the decellularization process. SEM examination and collagen fibril staining proved that the distribution of collagen fibrils for Tris-Trypsin-Triton treated samples was similar to the native samples, but SDS treated samples had a sparser distribution of collagen fibrils. Porosity assays further confirmed that SDS treated samples had larger porosity than Tris-Trypsin-Triton treated samples. Collagen content assay showed Tris-Trypsin-Triton treated and SDS treated samples had increased content compared with the native samples, which can be explained by the loss of other components during the decellularization process. GAG staining and content assay confirmed both Tris-Trypsin-Triton treated and SDS treated tumor sheets had decreased GAG components compared with their native counterparts. But Tris-Trypsin-Triton treated tumor sheets had more preservation of GAG components than the SDS treated counterparts. Cartmell and colleagues use SDS to decellularize rat tail tendons and observed some spaces between the collagen fibers but no evidence of denaturing of the native collagen [35]. Early studies conducted by Woods and colleagues presented that SDS treatment can cause significant loss of collagen and GAG components during decellularization [36], but previous studies conducted by Gratzer and colleagues showed SDS treatment led to structure but not content change [37]. The present study showed that the collagen and GAG structures and contents of Tris-Trypsin-Triton treated tumor sheets didn't change greatly, but SDS treated tumor sheets had altered collagen fibril structure and decreased GAG structure and content. Both ECM components and porosities play significant roles in cell proliferation and repopulation for tissue engineering. For SDS treatment, larger porosity has the potential to promote cell proliferation and infiltration [38], [39], but changed collagen structures and decreased GAG components have the possibility to limit cell repopulation [24], [37]. As a consequence, further cell culture study was needed to evaluate the performance of cell proliferation and repopulation in the Tris-Trypsin-Triton treated or SDS treated tumor sheets.

Mechanical properties play great roles in tumor cell differentiation and proliferation [11], [40], [41]. Mechanical properties are related to the porosities of tissue structures. 3D fiber-deposited scaffolds presented a decrease in dynamic stiffness and equilibrium modulus with an increasing porosity [42]. Stiffness of hydrogels was also found to be inversely correlated with pore size [43]. In this study, Tris-Trypsin-Triton treated and SDS treated tumor sheets had a reduced elastic modulus than their native counterparts, which was consistent with previous findings [44], [45]. Conversely, Williams and coworkers found that decellularized arteries had increased stiffness compared with the native arteries [46]. The collagen fibers of the adventitia were crimped for native arteries but became uncrimped after decellularization. Uncrimping of the collagen fibers and increasing of fiber mobility were thought to play significant roles for the increased stiffness. However, tumor sheets in this study had different structures compared with the arteries. The collagen fibers at pre- and post-decellularization were uncrimped. Additionally, solid tumors have a cell-rich structure, and both tumor cells and mesenchymal cells may increase stiffness of tumor scaffolds. Loss of these cells will lead to a reduction of stiffness.

Cell viability assays partly reflect the proliferation rate of cultured cells. The different cell viability observed between 2D and different 3D scaffolds can be related to the diffusion-limitations of 3D cultural environments [47]. Solid tumors often have microenvironments whereby cells at the centre are hypoxic, receiving less nutrients and oxygen than at the periphery [48]. The proliferation rate of tumor cells *in vivo* was much slower than *in vitro* 2D monolayer culture. Figure 5A demonstrated cells cultured onto Tris-Trypsin-Triton treated tumor sheets, SDS treated tumor sheets and Matrigel scaffolds had a reduced proliferation rate in comparison to 2D monolayer culture, which is in accordance with the performance of solid tumors *in vivo*. Further light microscopy and live/dead cell staining confirmed the cell proliferation profile found in Figure 5A. Light microscopy examination for the two acellular scaffolds in Figure 6C and 6D showed the matrices became optically opaque after a few days of culture. This confirmed that the light transmittance of scaffolds gradually decreased over time. Decrease of transmittance demonstrated increase of cell proliferation and repopulation because cells and their protein products hindered the transmittance of light through the scaffolds [6]. CFDA-SE/PI double fluorescence staining showed the number of dead cells gradually increased from day 7 to day 13, demonstrating denutrition and cell-cell inhibition for an extended culture time. Since cell proliferation at 13-day culture was similar to at 10-day culture of the four groups, the 10th day was chosen as the time point for growth factor expression assay. Particularly, the MCTS-like structure appeared when cells were cultured in Tris-Trypsin-Triton treated sheets at 13-day culture, which mimicked the cell clusters of solid tumors *in vivo*. Moreover, more cell numbers and deeper infiltration distances were detected for the Tris-Trypsin-Triton treated tumor sheets. It is different from the “growth on top” performance for the Matrigel scaffolds in Figure 6B and 7B. As a result, these acellular matrices have the potential to be used to investigate hypoxia, angiogenesis, metastasis mechanism and tumor drug resistance of solid tumor cells in the future.

IL-8, bFGF and VEGF have significant roles in tumor vascularization, and their expression is regulated by both cell-ECM interactions and 3D tumor cell culture conditions [6], [49], [50]. Growth factor secretion by MCF-7 cells was found to be up-regulated in the 3D culture group. In particular, 3D growth on decellularized matrix prepared using Tris-Trypsin-Triton treatment resulted in the most IL-8 secretion. It is in accordance with previous work by Fischbach and colleagues that 3D culture led to obviously enhanced IL-8 secretion but only modestly modulated VEGF secretion [6]. Combined with the cell proliferation results, we conclude that Tris-Trypsin-Triton treated tumor sheets are superior to SDS treated tumor sheets as 3D scaffolds for tumor engineering.

Tests have been performed using A549 cells, human colorectal adenocarcinoma SW-480 cells and human esophageal squamous cell carcinoma KYSE-510 cells to broaden the scope and general applicability of the study. It is interesting that they did not all behave in the same way. A549, SW-480 and MCF-7 cells had similar behavior when cultured within the Tris-Trypsin-Triton treated tumor sheets. This meant the decellularized matrix of a pulmonary adenocarcinoma cell line supported the repopulation of a pulmonary adenocarcinoma cell line, as well as the repopulation of a colorectal or breast cancer cell line. However, KYSE-510 cells had a slower cell repopulation rate than other tumor cells. It should not be taken for granted that rate of repopulation of the scaffold need necessarily correspond to monolayer culture growth rate, so this observation remains worthy of mention as is the fact that the relatively fast tumor growth rate of A549, SW480 and MCF-7 cells presented a particular advantage over KYSE-510 cells.

With regard to alternative 3D culture models, decellularized lung scaffolds were developed and used for tissue engineering or testing the growth of lung cancer cells [51]–[53]. This alternative successful use of a natural scaffold to mimic *in vivo* biological processes involved in human lung cancer, was nonetheless more complex and expensive than our procedures with unknown efficacy for supporting the 3D culture of cancer cells from other organs. Alternatively, synthetic scaffolds, such as functionalized PEG-based scaffolds with a potential to mimic the natural microenvironment, have been used to study epithelial morphogenesis and epithelial–mesenchymal transition (EMT) in a lung adenocarcinoma model [13]. Biochemistry and mechanical properties of the synthetic matrices were tunable. Such acellular matrices have similar microenvironment to native tumors, serving as a platform for study of *in vivo*-like behavior of tumor cells. Natural and synthetic scaffolds present complementary approaches, each with distinct advantages regarding modification of matrix biochemistry and mechanics.

In conclusion, this study shows that tumor sheets decellularized by Tris-Trypsin-Triton multi-step treatment or SDS treatment had a complete cellular removal, with part loss of ECM components, increase of porosity and decrease of mechanical stiffness. Tris-Trypsin-Triton was better than SDS treatment of tumor sheets for preserving ECM structures and components, maintaining optimal 3D tumor cell proliferation and repopulation with most IL-8 secretion. These results indicate the advantages of the Tris-Trypsin-Triton multi-step decellularized tumor ECM as a suitable 3D scaffold for tumor engineering. Further work is necessary to explore the malignant phenotype of tumor cells in 3D culture, hypoxia with an extended culture time, and cellular responses to chemotherapeutic agents.

## Supporting Information

Figure S1 **Cell viability detected by MTT assay for A549 (A), SW-480 (B) and KYSE-510 cells (C) cultured within 2D and Tris-Trypsin-Triton group over time.** Graph represents mean ± SD of three independent experiments (n = 5).
(TIF)

Figure S2 **Growth curve of KYSE-510 cell derived tumors.** Human esophageal squamous cell carcinoma KYSE-510 cells were implanted in severe combined immunodeficiency (SCID) mice to form solid tumors (n = 6). Tumor volumes were about 20 mm^3^ at 30-day implantation, 130 mm^3^ at 60-day implantation and 600 mm^3^ at 90-day implantation.
(TIF)
