# Supplementary figures and images for: Development of an Acellular Tumor Extracellular Matrix as a Three-Dimensional Scaffold for Tumor Engineering
Source: PLoS One. 2014 Jul 29;9(7):e103672. doi: 10.1371/journal.pone.0103672 (PMC4114977; doi:10.1371/journal.pone.0103672)

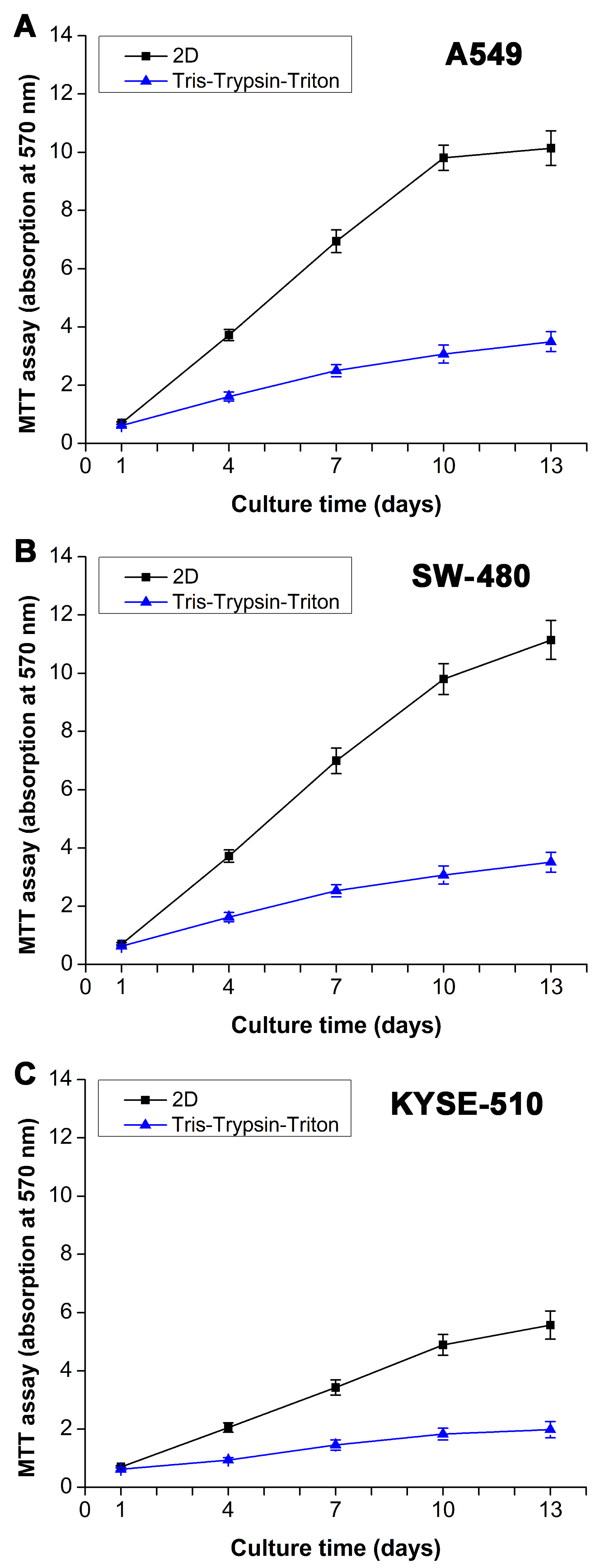

Supplement: Figure S1 — Cell viability detected by MTT assay for A549 (A), SW-480 (B) and KYSE-510 cells (C) cultured within 2D and Tris-Trypsin-Triton group over time. Graph represents mean ± SD of three independent experiments (n = 5). (TIF) [file pone.0103672.s001.tif]

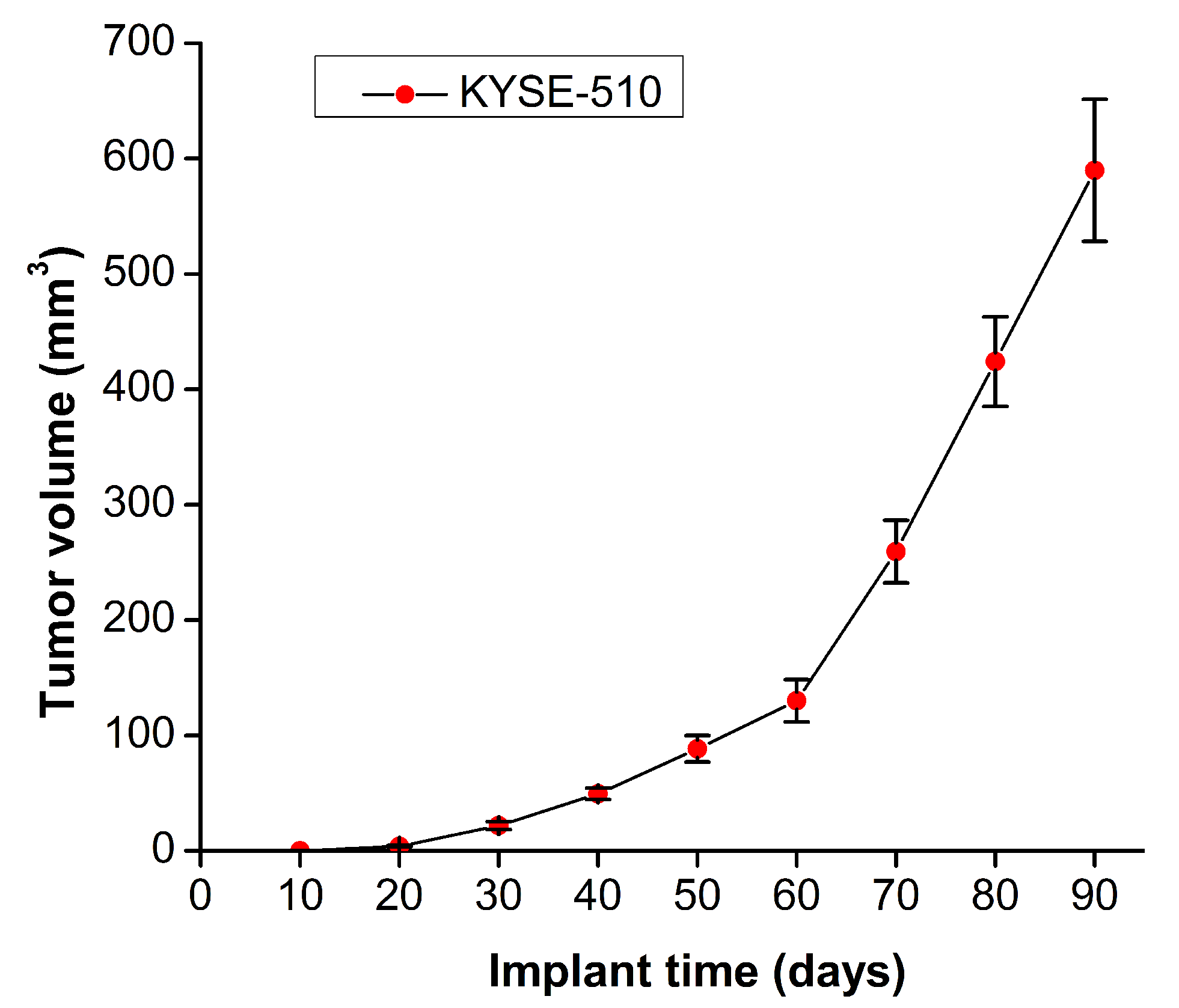

Supplement: Figure S2 — Growth curve of KYSE-510 cell derived tumors. Human esophageal squamous cell carcinoma KYSE-510 cells were implanted in severe combined immunodeficiency (SCID) mice to form solid tumors (n = 6). Tumor volumes were about 20 mm3 at 30-day implantation, 130 mm3 at 60-day implantation and 600 mm3 at 90-day implantation. (TIF) [file pone.0103672.s002.tif]
